# Diversity in autoimmunity against retinal, neuronal, and axonal antigens in acquired neuro-retinopathy

**DOI:** 10.1007/s12348-011-0028-8

**Published:** 2011-07-10

**Authors:** Grazyna Adamus, Lori Brown, Jade Schiffman, Alessandro Iannaccone

**Affiliations:** 1 Casey Eye Institute, Oregon Health and Science University, Portland, OR USA; 2 Neuro-Ophthalmology, University of Texas M. D. Anderson Cancer Center, Houston, TX USA; 3 Hamilton Eye Institute, University of Tennessee Health Science Center, Memphis, TN USA; 4 Casey Eye Institute, BRB L467AD, Oregon Health and Science University, 3181 SW Sam Jackson Pk Rd, Portland, OR 97239 USA

**Keywords:** Autoimmunity, Retinopathy, Optic nerve, Neuropathy, Autoantibodies, Retina, Immunofluorescence, Western blotting, Retinal degeneration

## Abstract

**Purpose:**

Autoimmune retinopathies and optic neuropathies are complex disorders of the retina and the optic nerve, in which patients develop autoantibodies (AAbs) against retinal and optic nerve proteins. Autoimmunity might significantly influence the outcome of retinal and optic nerve degenerative process but the pathogenic process is not fully elucidated. To better understand the role of AAbs in pathogenicity of these suspected autoimmune visual disorders, we focused on unique AAbs specificities associated with the syndrome to identify their antigenic targets in the optic nerve and retina.

**Methods:**

Serum samples were obtained from patients, whose visual disorders were potentially autoimmune in nature, including patients with cancer with possible paraneoplastic syndrome. Autoantibodies were tested against human optic nerve and retinal antigens for specificity by Western blotting and immunofluorescence.

**Results:**

Out of 209 tested for anti-optic nerve autoantibodies, 55% showed specific neuronal autoantibodies. The repertoire of anti-optic nerve autoantibodies often differed from anti-retinal antibodies. The major antigenic targets for these antibodies could be divided into four groups. Autoantibodies specific to classical glycolytic enzymes involved in energy production (α and γ enolases, glyceraldehyde 3-phosphate dehydrogenase) also reacted with retinal antigens. Autoantibodies targeted neuronal-specific myelin proteins (MBP, MOG), aquaporin 4, and collapsing response mediator protein 5 reacted with optic nerve antigens. They showed immunostaining of axons and myelin in the optic nerve as determined by double immunofluorescence.

**Conclusion:**

We identified novel neuronal autoantigens not previously known to be associated with acquired autoimmune retinopathy and optic neuropathy. Knowledge of the full autoantibody repertoire perpetuating this syndrome is an important first requirement in increasing our understanding of the autoimmune process to facilitate better diagnosis, prognosis, and treatment.

## Introduction

Presence of autoantibodies (AAbs) specific against antigens present in the retina is the hallmark of autoimmune process in cancer-associated retinopathy (CAR) and autoimmune retinopathy (AR) [1, 2]. Patients diagnosed with paraneoplastic or non-paraneoplastic retinopathy often present with optic nerve problems not related to glaucoma. Serum antibodies against neuronal antigens have been detected in a wide variety of neurological disorders but anti-optic nerve AAbs has not been extensively studied. Autoimmune retinopathies and optic neuropathies are complex disorders of the retina and the optic nerve, in which patients develop AAbs against retinal and optic nerve proteins [3]. Dependent on whether the syndrome is targeting the retina, optic nerve, or both, the pathogenesis of these syndromes are poorly elucidated and may be characterized by progressive loss of visual acuity and/or visual field sensitivity, loss photoreceptors and/or retinal ganglion cells (RGCs) and their axons, and optic nerve head changes, such as pallor, hyperemia, edema, swelling, or dropout of the retinal nerve fiber layer (RNFL) by optic coherence tomography (OCT) imaging [4–8].

Thus far, most studies have focused on AAbs in glaucoma and neuronal loss due to the loss of RGCs [9, 10]. The perceived pathology of autoimmune optic neuropathy and glaucoma is different, although both might have a similar underlying autoimmune cause [6, 11]. It is important to point out that the immune response might significantly influence the outcome of retinal and optic nerve degenerative process. For example, AAbs can exert their pathogenic effects if they gain access from the periphery to the central nervous system (CNS)/eye when the integrity of the blood barriers is compromised. Because there is little known about pathogenicity of retinopathy and associated optic neuropathy, we undertook a first line of investigation focusing on unique AAbs specificities associated with the syndrome that is thought to be autoimmune in nature, including patients with cancer with potential paraneoplastic syndrome. These patients had symptoms of optic nerve and/or retinal dysfunction, some of which have been found to have electrophysiological evidence of retinal process, or other findings to suggest an optic nerve process. However, work-up did not reveal a definite known disorder. In this patient population, we found autoantibodies that target antigens present in the optic nerve and retina, may affect the outcome of the visual dysfunction. The identity of antigenic targets that those AAbs recognize in the optic nerve will allow for a better understanding of the molecular mechanism of RNFL and axonal loss during optic neuropathies. Our study shows that these patients have unique AAbs against neuronal antigens often different from the retinal antigens.

## Materials and methods

### Patients

Patient's sera were acquired through the Ocular Immunology Laboratory through retrospective serological evaluation for anti-retinal autoantibodies. The study has been approved by the OHSU Institutional Review Board.

### Western blotting

Human retinal proteins were extracted from the retina or optic nerve with 2% octyl glucoside in phosphate buffer, pH 7.2 and 10-μg protein was used for electrophoresis using Bio-Rad Criterion gels following by transfer to PVDF membrane [2]. As positive controls, we used monoclonal antibodies against α-enolase and γ-enolase diluted 1:2,000. A negative control contained secondary antibodies only. The reactivity with a suspected known protein was confirmed by an additional Western blotting using an appropriate purified protein. Also, we used brain extract prepared by the same method as was used to extract retinal proteins to confirm reactivity with neuronal antigens.

### Fluorescent immunohistochemistry

Rat eye posterior globe containing the whole retina and optic nerve was fixed in 4% paraformaldehyde for 1 h followed by 30% sucrose infiltration overnight and the globe was frozen in Tissue-Tek optimal cutting temperature compound. Ten-micron cryosections were prepared for incubation with human serum. Briefly, sections were blocked with 10% normal goat serum with 1% bovine serum albumin for 60 min followed by incubation with patient's serum overnight. After washing, fluorescent anti-human IgG labeled with FITC or biotin-labeled anti-human IgG followed by Texas Red labeled to streptavidin were added for 1 h. The mounting reagent, which inhibited fluorescence quenching and contained DAPI for nuclear staining was used to seal the sections. The labeling was evaluated in a Leica DM5000 fluorescent microscope and pseudocolor images were acquired for analysis. A negative control contained secondary antibodies only.

For double staining we used biotinylated anti-human IgG followed by Streptavidin conjugated to Texas Red or antibodies specific to cell markers at 1:100 dilutions: neuronal fibers (RT97) (Chemicon), glial fibrillary acidic protein (GFAP) (BD Pharmingen) for astrocytes and cyclic nucleotide phosphodiesterase (CNPase) (Thermo Scientific) and myelin oligodendrocyte glycoprotein (MOG) (Abnova) for oligodendrocytes followed by the appropriated secondary antibodies conjugated to Alexa Fluor 488. After incubation with antibodies, tissue sections were washed and mounted with DAPI. The acquired images were merged using Photoshop software to show specific cellular labeling.

## Results

### General characteristics of patients

**Fig. 1:**
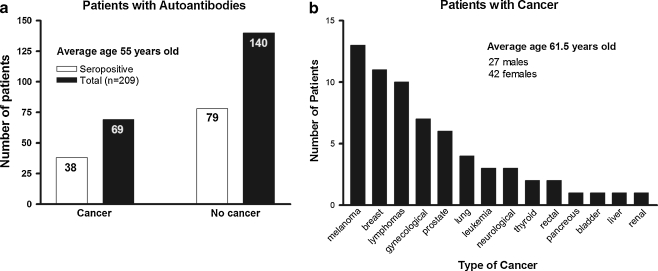
Demographics of 209 patients with autoimmune retinopathy and autoimmune optic neuropathy in the study: **a** Seropositive patients with anti-optic nerve autoantibodies in patients with cancer and without cancer. **b** Distribution of cancers in 69 patients with paraneoplastic syndromes

Our cohort of 209 patients presented painless and progressive, most commonly acute or subacute visual loss, loss of visual fields, defects in color vision, optic nerve head changes on ophthalmoscopy, usually decreased retinal function on electroretinigraphy (ERG), delayed visual evoked potentials (VEPs), in some thinning of the retina and RNFL changes on OCT imaging, and showed the presence of serum antibodies against optic nerve proteins in addition to anti-retinal AAbs. Optic nerve head changes, including: marked hyperemic swelling; RNFL thickening, thinning, or both; disc pallor;, cupping of the optic nerve head; and/or frank optic nerve atrophy was reported in 51 patients. Of the 209 patients with average age 55 years old (ranged from 18 to 88 years old), 116 patients (55.5%) had AAbs against retinal and optic nerve antigens (Fig. 1a). The ratio of male to female was almost equal (1:1.5). Sixty nine of 116 patients (42 women and 27 men) of average age 61.5 years old had different types of cancer. The most common cancers associated with the syndrome were breast cancers (13 patients) and melanomas (11 patients) (Fig. 1b). Thirty eight out 69 paraneoplastic patients had AAbs against optic nerve proteins that often *differed* in specificity from anti-retinal proteins and targeted autoantigens specific for the optic nerve. Table 1 shows AAb testing results from 30 paraneoplastic patients with systemic cancers; 7 patients with intraocular cancer were not included in the table.

**Table 1:** Paraneoplastic neuro-retinopathy patients with anti-optic nerve and anti-retinal AAbs

Number	Sex	Age	Diagnosed cancer	Onset of cancer	Onset of visual symptoms	Reported optic nerve findings	Anti-optic nerve AAbs	Anti-retinal AAbs
1	F	43	Brain venous angioma	2006	2004		46 k	46 k
2	F	43	Breast			Apparent temporal pallor	27 k, 35 k	27 k
3	F	50	Breast	2005	2007	Bilateral temporal optic disc pallor and juxtapapillary atrophic changes OD > OS	46 k	46 k
4	F	59	Breast	1999	2006	Glaucoma	24 k,36 k, 50 k, 70 k	46 k, 62 k, 70 k, 112 k
5	F	55	Breast			Optic neuropathy	46 k, 82 k	46, 66 k, 82 k
6	F	68	Breast			Sequential episodes of optic neuropathies	30, 42 k low	negative
7	F	58	Breast				23 k, 27 k	23 k, 25 k, 32 k, 33 k
8	F	60	Breast		2008		62 k	35 k,38 k low
9	F	47	Breast metastatic to brain			Congenital optic nerve change with tilting OS,	30 k, 36 k	46 k
10	F	56	Cervix	1983	2009	Slightly enlarged cup to disc ratio bilaterally	36 k, 42 k, 96 k	30 k
11	F	58	Cervix				46 k	
12	M	64	Colorectal	2004	2008		23 k	40 k, 44 k, 46 k, 60 k
13	F	71	Endometrium metastatic				46 k	46 k high
14	F	61	Glioblastoma			Homonymous hemianopsia OD, hemi between the macula and optic nerve	35 k low	35 k low
15	M	43	Malignant hepatoma	2006	2005?	Optic atrophy	33 k	33 k
16	F	65	Lung	2008	2006		7 proteins 35–50 k	28 k, 46 k
17	F	78	Lung non-small cell carcinoma			Holes in both capsules	30 k	30 k
18	F	55	Lymphoma CNS		2006		70 k	70 k
19	F	83	Melanoma			Optic neuropathy	28 k	47 k, 70 k, 112 k
20	F	79	Melanoma				46 k, 45 k	46 k
21	M	61	Melanoma				30 k	30 k
22	F	72	Ovarian				46 k	35 k, 46 k
23	M	77	Prostate	1996	2003	RNFL slightly thinned temporally OD;	61 k, 62 k	30 k, 67 k, 112 k
24	M	79	Prostate	2005	2000		41 k	negative
25	M	85	Prostate and adrenal adenoma	2002			24–25 k	negative
26	M	77	Prostate and skin cancers	2001	2005		24–26 k	negative
27	F	61	Rectal	2008	2003	Very enlarged blind spots OU	46 k	46 k
28	F	35	Renal	~2008			46 k	30 k, 46 k, 90 k, 140 k
29	F	70	Thyroid	2006		Vertical optic nerve head cupping OS > OD, in OS with peripapillary atrophy	90 k	49–50 k, 90 k
30	M	72	Thyroid benign	2004	~1973		36 k, 54 k	27 k, 30 k, 33 k

*k* kilodaltons; *OS* left eye, *OD* right eye

Since the presence of AAbs against optic nerve antigens has not been previously tested in healthy individuals, we also analyzed 56 normal sera (32 females of average age 47 years and 24 males of average age 45 years) for anti-optic nerve AAbs by Western blotting. The vast majority of normal sera, showed no or low reactivity with optic nerve proteins. Only four out of 56 sera (7%) weekly reacted with optic nerve proteins (35, 46, 62 kDa) as determined by Western blotting (not shown).

### Anti-optic nerve autoantibodies

**Fig. 2:**
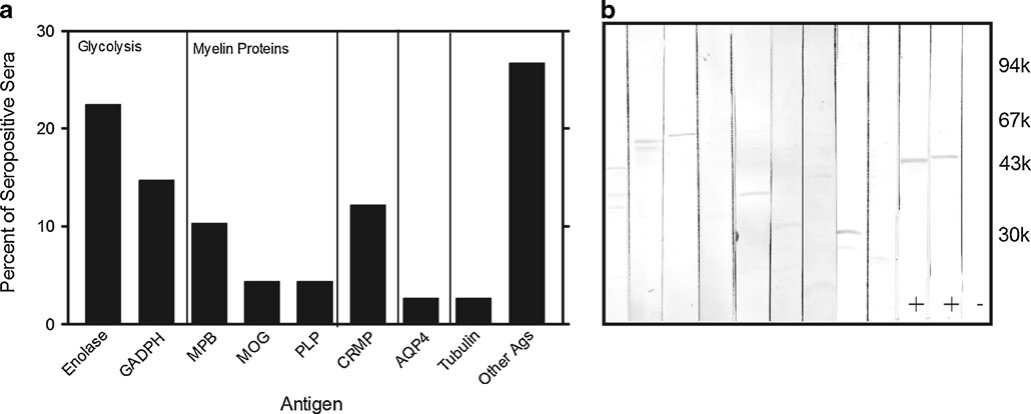
Western blot analysis of autoantibodies against optic nerve present in patients in the studies: **a** *Bar graph* showing a percent of patients' sera reacting with the following autoantigens: *Enolase*, glyceraldehyde 3-phosphate dehydrogenase (*GAPDH*), myelin basic protein (*MBP*), oligodendrocyte-myelin glycoprotein (*MOG*), or proteolipid protein (*PLP*), *CRMP*-5, aquaporin-4 (*AQP4*), *Tubulin*, and other unidentified autoantigens in the optic nerve. **b**. A representative blot for nine patients, two positive controls marked by (*positive sign*) correspond to neuron specific enolase, and α-enolase; (*negative sign*) negative control, no patient's serum

**Fig. 3:**
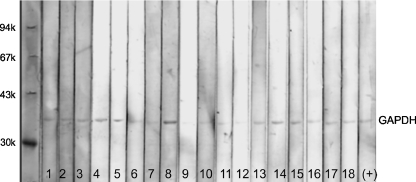
Immunoreactivity of 18 representative patients' sera and control specific antibodies with 1-μg purified human GAPDH proteins was loaded on the blot

Initially, the patients' sera were tested for anti-retinal and anti-optic nerve autoantibodies by Western blotting and we found that antibodies against both tissue antigens coexist in the serum (Fig. 2). In this study, we focused on identification of optic nerve autoantigens that immunoreacted with serum AAbs. The highest number of serum AAbs (23 patients) bound to enzymatic proteins, including both α and γ (or NSE, neuron specific enolase) enolases (46 kDa). In addition, patient AAbs frequently targeted retinal and optic nerve antigen of apparent molecular weight of 36 kDa that was further analyzed for molecular identify by immunoprecipitation. This new antigen was identified by MS/MS analysis as glyceraldehyde 3-phosphate dehydrogenase (GAPDH), also a glycolytic enzyme that converts glyceraldehyde 3-phosphate to D-glycerate 1,3-bisphosphate. Figure 3 shows reactivity of 18 different sera with purified human GAPDH protein on the blot. Some patients recognized another glycolytic enzyme, aldolase. Forty sera had antibodies that recognized low molecular weight proteins associated with CNS myelin such as myelin basic protein (MBP, 33 kDa), MOG (28 kDa) or proteolipid protein, PLP (30 kDa). These anti-neuronal antibodies bound to the optic nerve and brain proteins but not to retinal antigens, which is in agreement with the unique localization of those myelin proteins in CNS tissues, including optic nerve (not shown). Also, several patients reacted with 62-kDa protein that had the same molecular weight as collapsin response-mediated protein 5 (CRMP5). Previously published studies showed anti-CRMP5 antibodies were associated with paraneoplastic optic neuritis [12]. Four patients reacted with 40-kDa optic nerve protein, which corresponded to our control antibodies against aquaporin 4 (AQP4), a water channel protein usually associated with neuromyelitis optica [13].

### Astrocytic and neuronal immunoreactivity in the optic nerve

To further confirm the immunoreactivity with optic nerve proteins and to identify cellular targets for these seropositive AAbs in relation to autoimmune optic neuropathy, we carried out a series of double-immunofluorescent labeling studies using rat optic nerve thin cryosections. The patients' sera were co-incubated with specific antibodies against astrocytes (GAFP), neuronal fibers (RT97), and oligodendrocytes (CNPase and MOG), aquaporin 4 (AQP4), NSE, and CRMP-5 in double-staining experiments. No oligodendrocyte-specific labeling was detected in the retina (not shown). GFAP immunolabeled the RGC layer and RT-97 labeled the neuronal axons of the normal rat retina. All those antigens were present in the optic nerve. No staining was observed with control sera whereas groups of seropositive sera showed similar labeling patterns.

**Fig. 4:**
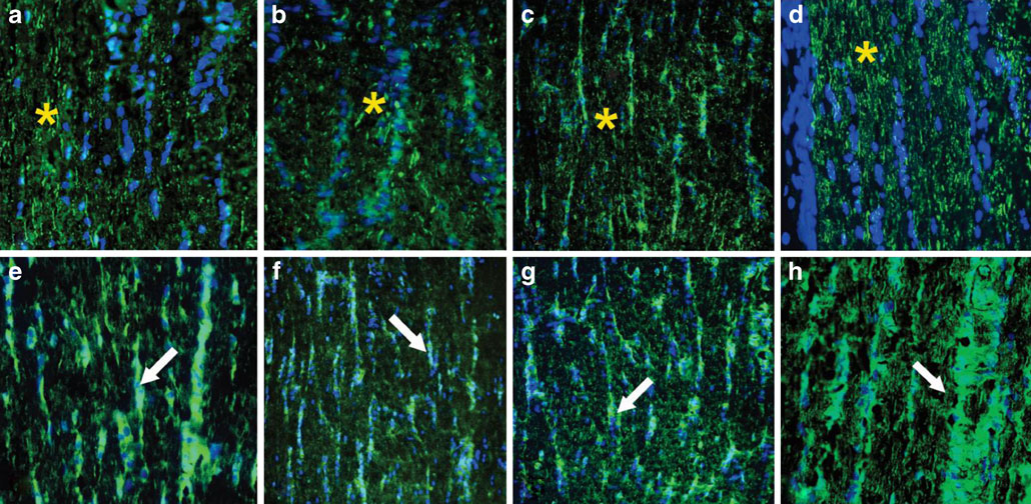
Immunolabeling patterns by patients' autoantibodies on the longitudinal sections of the rat optic nerve. Representative immunofluorescent labeling for 3(**a, b, c**) sera shows labeling of axons similar to that of the axonal marker RT97 (**d**) indicated by *asterisks* (*top pictures*). *Bottom pictures* show other three representative sera (**e, f, g**) that labeled astrocytic columns (*arrows*) in the optic nerve in a similar pattern to that of the astrocyte marker GFAP (**h);** *green* FITC for antibodies; *blue* DAPI for nuclei

**Fig. 5:**
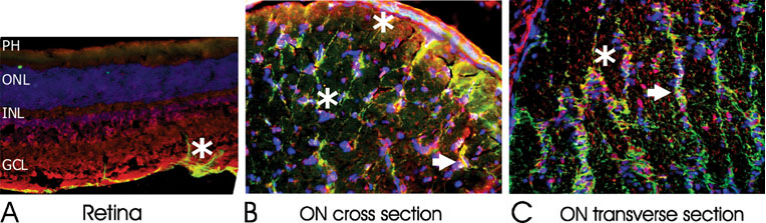
Double-immunofluorescent labeling in the rat retina (**a)** and optic nerve (**b, c**) with anti-GFAP antibodies as marker for astrocytes showing that patient's autoantibodies are predominantly anti-astrocytic (*asterisks* and *arrows*) : serum (*red*), GFAP (*green*), and colocalization (*yellow*), nuclear labeling with DAPI (*blue*); *ON* optic nerve

**Fig. 6:**
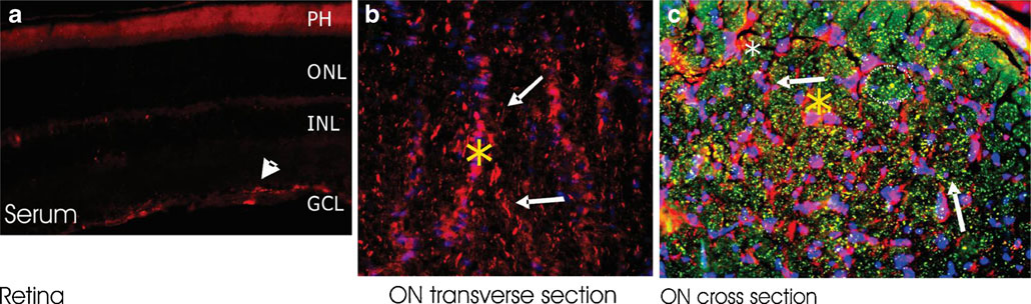
Double-immunofluorescent labeling patient's autoantibodies showing labeling of neuronal axons in the retina (**a**) and transverse (**b**) and cross section of optic nerve (**c**). serum (*red*), RT97 (*green*), and colocalization in **c** (*yellow*); nuclear labeling with DAPI (*blue*). *Note* that axons were compartmentalized into distinct bundles (*circled* in **c**)

Surprisingly, seropositive AAbs tested revealed strong immunolabeling of the optic nerve. Most sera labeled the optic nerve in two different patterns—they showed axonal or glial staining. Figure 4 shows an immunolabeling of axonal fibers (A–C) and glial columns (D–E) by representative sera in a similar pattern to that of specific marker antibodies for those cellular structures such as axonal staining with RT97 antibodies and glial staining by anti-GFAP antibodies, respectively. The labeling of astrocytes in the RGC layer and optic nerve confirmed by GFAP co-labeling implies that these AAbs recognize antigens located in astrocytes. For instance, as shown in Fig. 5, strong immunolabeling of astrocytes was present (Fig. 5b,c) on cross- and transverse sections of the optic nerve by AAbs that showed reactivity with optic nerve proteins of molecular weight 30 and 36 kDa (GAPDH) on the blot. We observe that number of sera-labeled RGC axon bundles in the nerve fiber layer, the lamina cribrosa, and the retrobulbar optic nerve. A representative double-immunofluorescent labeling for patient's serum and RT97 antibodies, an axonal marker, confirmed the AAbs specificity for neuronal axons (Fig. 6). AAbs-positively immunolabeled cell processes organized in long trails in the optic nerve (asterisks).

**Fig. 7:**
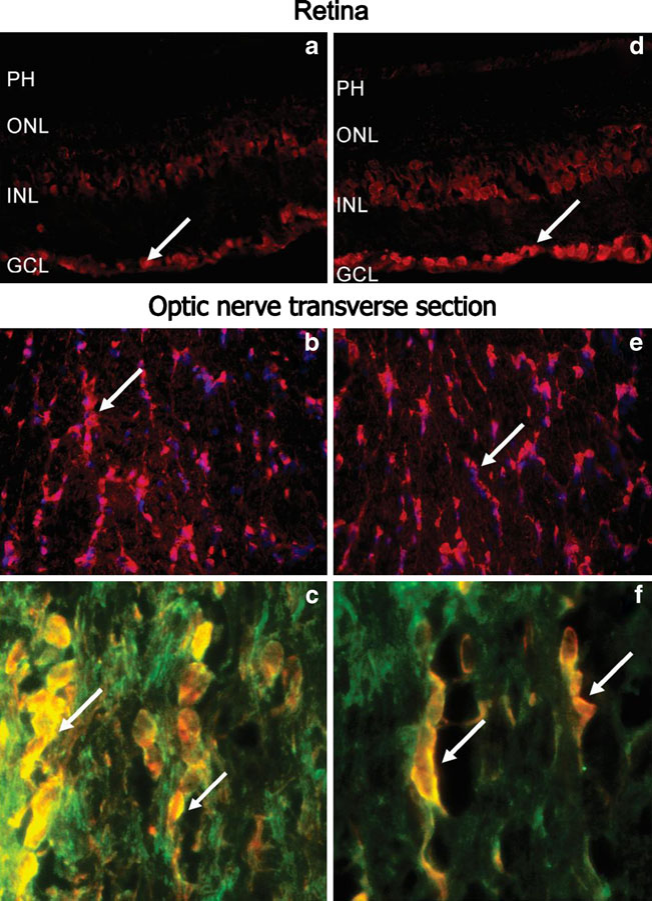
Immunofluorescent labeling of the retina and optic nerve with two representative patients sera that labeled cells in ganglion and inner nuclear layer in the retina (**a, d**) but oligodendrocytes in the optic nerve (**b, e**; *arrows*); in double-immunofluorescence labeling autoantibody immunoreactivity overlapped with anti-MOG antibodies, a marker for oligodendrocytes (**c, e**); serum (*red*), MOG (*green*), and colocalization (*yellow*), nuclear labeling with DAPI (*blue*)

The different immunolabeling pattern is represented by the strong staining of cell bodies and their dendrites in the optic nerve. Figure 7 shows representative pictures for two patient's sera that have AAbs against an optic nerve 42-kDa protein. In the retina, these AAbs labeled RGCs and cells in the inner nuclear layer (Fig. 7a, d). The optic nerve cellular labeling strongly suggested their binding to oligodendrocytes (Fig. 7b, e). Consequently, we double labeled the optic nerve with patient's serum and anti-MOG antibodies as marker for oligodendrocytes. As it can be seen on the transverse ON sections, the patients' AAbs bound to oligodendrocyte cell membranes and processes in the same manner as did anti-MOG antibodies (strong yellow color on double-labeling experiment in Fig. 7c, f), confirming specificity of antibodies to myelin protein.

**Fig. 8:**
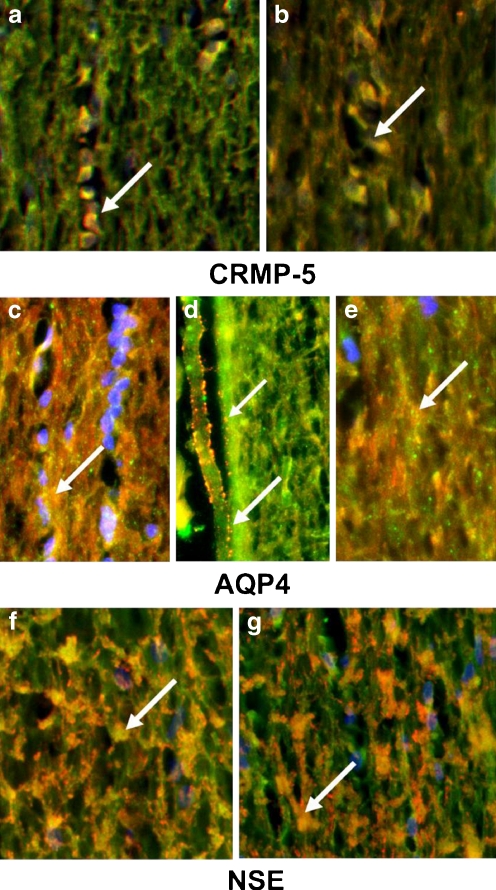
Representative patient's sera immunoreactivity with the optic nerve. Longitudinal section of the rat optic nerve double-labeled representative patients' sera (*green*) and one of the following control antibodies: **a, b** with anti-CRMP-5 (*red*), **c–e** anti-APQ4 (*red)* and **f, g** anti-NSE antibodies (*red*); colocalization (*yellow*) and nuclear labeling with DAPI (*blue*); *arrowheads* point at the reactive cellular structures

The presence of AAbs in the patients' sera against 62-kDa protein suggested reactivity with CRMP-5 protein. Double immunofluorescence of the optic nerve showed that anti-62-kDa-specific sera labeled oligodendrocyte soma (cytoplasm) and processes close to myelin in a similar pattern to specific anti-CRMP-5 antibodies (Fig. 8a,b). Other AAbs immunolabeled astrocytes and showed a distinct punctate immunoreactivity on the cell surface and blood vessels (Fig. 8c–e) indicative of AQP4 labeling. AQP4 is expressed on astrocytes. In double-staining experiments, we co-localized three patients' sera with anti-AQP4 antibodies in the optic nerve (yellow merged color). A number of sera recognized NSE when incubated with optic nerve proteins on the blot and those sera stained predominantly the cytoplasm of oligodendrocytes in the tissue (Fig. 8f, g).

## Discussion

Autoimmunity to retinal, neuronal, and axonal antigens may play a role in inducing cellular degeneration. The specific AAbs against retinal antigens are most commonly associated with CAR and AR but their pathogenic effects were not fully established [14]. The current studies showed that serum anti-optic nerve AAbs are also present in more than 50% patients tested in patients with either CAR or AR. The major optic nerve antigenic targets for patients AAbs described in these studies can be divided into four categories as follows: (1) classical glycolytic enzymes involved in energy production, including α and γ enolases, glyceraldehyde 3-phosphate dehydrogenase, which also reacted with retinal antigens; (2) neuronal-specific myelin proteins; (3) CRMP5; (4) AQP4. Also other antibodies were present but their antigens have not yet been discovered and are under investigation. The newly identified AAbs labeled optic nerve components such as axons, astrocytes, and oligodendrocytes. Although their role in the pathogenicity of the optic neuropathy affecting these patients needs to be evaluated further, we believe that these unique anti-optic nerve AAbs can be valuable disease markers. We observed that the AAb presence correlated with various clinical (ophthalmoscopic changes at or around the disc), imaging (thinning or, in fact, swollen RNFL), and functional findings presented by affected patients, not usually seen in retinopathies. Moreover, distinct autoantigens may become novel therapeutic targets in disease, e.g., blocking GAPDH-mediated cell death in pathological conditions. Therefore, the identity of cellular targets is important in better understanding the etiology of autoimmune retinopathy associated with optic neuropathies, whether with or without retinopathy, and for developing better treatments. Our research was solely intended to identity of anti-optic nerve AAbs targets because this area was not explored before. The future studies will evaluate their responsiveness to immunosuppressive treatments for neuro-retinopathy targeting reducing the antibody titers on follow-up testing and clinical response to therapy.

The definition of the autoimmune neuro-retinopathy is not well defined. Autoimmune optic neuropathy (AON) was first described by Dutton et al. as recurrent episodes of optic neuropathy with AAbs but as a different entity distinct from demyelinating optic neuritis [15]. In general, AON has been characterized by chronically progressive or recurrent visual loss associated with serological evidence of autoimmunity and without a defined systemic autoimmune disorder [4]. Later, autoimmune-related retinopathy and optic neuropathy (ARRON) has been defined by the Keltner’s group as a syndrome that is characterized by visual loss and often the presence of antibodies against retinal or optic nerve antigens in the absence of cancer [16]. Our patients suffer from retinopathy and optic neuropathy, more than half had both anti-retinal and anti-optic nerve autoantibodies, and third of them has been diagnosed with cancer at the time of testing for antibodies. We recommend the following unique measurements that help improve the diagnosis for optic neuropathy in the context of retinopathy: delay VEPs, pattern of visual fields loss that is not clearly consistent with retinopathy, attenuated and/or thickened RNFL as measured by OCT imaging criteria, compromised photopic negative response (PhNR) of the photopic flash ERG, which is mediated by RGCs, and the presence of AAbs, some of which may label RGCs in the tissue. Our research identified new AAbs specific against optic nerve antigens not previously known in autoimmune neuropathy associated with retinopathy, suggesting the autoimmune nature of the syndrome also at the optic nerve level. If pathogenic, AAbs binding to proteins in optic nerve neurons may lead to disruption of protein function, apoptosis of cells, as well as inhibiting enzymes that preserve neuronal glycolytic activity and neuronal integrity.

A high prevalence of various AAbs in patients with neuro-retinopathy suggests a polyclonal activation of the humoral immune system. It is not clear what the role of each AAb is in autoimmune optic neuropathy and whether the AAbs contribute to pathogenicity at all. We hypothesize that autoimmune damage to the retina and optic nerve may be caused directly by the AAbs, or indirectly by a non-immune cause that contributes to tissues damage but may activate the secondary immune responses as a result of neuronal degeneration. During pathogenic processes, AAbs could bind to neuronal proteins in optic nerve and retina, inhibiting enzymes that preserve neuronal glycolytic activity and neuronal integrity, which may ultimately lead to apoptosis of those cells [17]. Thus, AAbs may be directly involved in the induction of pathogenic processes or could be related to the progression over time of ongoing pathological processes in the retina and optic nerve. In the latter, specific AAbs related to specific phenotype could be considered as biomarkers related to disease. We identified AAbs that belong to the group of neuronal glycolytic enzymes such as GAPDH (glyceraldehyde 3-phosphate dehydrogenase) [18] and neuronal-specific and non-neuronal enolases [19]. These are multifunctional proteins that are all expressed intracellularly but also on the neuronal cell surface [20, 21]. They are involved in energy metabolism, cell signaling, and synaptic neurotransmission [22]. Although these AAbs can be present in normal controls, they were present significantly more frequently in the sera of retinopathy patients [23]. The biological significance of anti-GADPH must be related to the roles of GADPH as a multifunctional enzyme that is involved in apoptosis, oxidative stress, activation of transcription, membrane fusion, and vesicle transport. GADPH is a cytosolic protein but can translocate to the nucleus in apoptotic processes. Interestingly, GADPH is present in large quantities in rod outer segments playing a role in the production of energy for these active cells [21]. Therefore, neuronal death induced during glycolysis inhibition involves calcium influx through NMDA receptors and calcium release from intracellular ER stores [17, 24]. Uncontrolled increases in intracellular calcium lets to apoptotic cell death. It has been suggested that GADPH plays a pathogenic role in human age-related neurodegenerative diseases, including Huntington's disease, Parkinson's disease, and Alzheimer's disease [25]. Neuronal damage related to aging and chronic neurodegenerative diseases has been suggested to be associated with decreased glucose metabolism due to AAb action [24, 26]. Such possibility exists because GADPH is a strong antigenic protein, especially during infections, which leads to elucidation of anti-GADPH AAbs [22] and similarity in the amino acid sequence of Streptococcal and human proteins explains a cross-reactive immune response against this protein [27]. Likewise, enolase and aldolase are also expressed on *Streptococcal pyogenes* and they too show significant identity between Streptococcal and human neuronal glycolytic enzymes (apart from aldolase C) [28].

Only a few of our patients have AAbs targeting the astrocytic water channel protein, AQP4. Anti-AQP4 AAbs has been found in a high percentage (~75%) of neuromyelitis optica patients (NMO-IgG), usually identified by immunofluorescence only [13], suggesting the importance of conformational epitopes, but also linear epitopes were later recognized in denatured protein [29]. The recognition of linear epitopes could explain the immunoreactivity discordances observed for some serum samples, which tested positive by immunocytochemistry but were negative by WB, and vice versa. NMO is a rare inflammatory demyelinating disease that selectively affects optic nerves and spinal cord, usually not seen in multiple sclerosis (MS) and other demyelinating syndromes of the CNS [30]. AAbs against AQP4, which is expressed in astrocytic endfeet at the blood brain barrier, are believed to induce damage to astrocytes [31]. The close contact of AAbs/AQP4 positive processes with oligodendrocytes and myelin tracts suggest that a bystander effect of AAbs-damaged astrocytes on oligodendrocytes might occur in the nervous tissues affected by autoimmune optic neuropathy.

Optic neuritis associated with anti-AQP4-Ab affected only females who had bilateral eye involvement, and all had severe visual impairment in the acute phase and delayed partial visual recovery [32]. Our patients with serum anti-AQP4 were male. Similar to our patients, NMO patients showed a predominant positive anti-MOG response and anti-MBP AAbs. There are some clinical features that overlap between autoimmune optic neuropathy and NMO [6]. Despite the limited number of samples, presence of such AAbs suggests a predominantly widespread acute immune activation, including a strong B-cell response [30]. However, the role of anti-AQP4 AAbs in neuro-retinopathy is not clear.

AAbs against CRMP5 were reported in paraneoplastic syndrome patients with optic neuropathy and retinopathy associated with the presence of vitreous and intrathecal cells [12]. The neurological syndromes associated with CRMP5 antibodies are very diverse (much like those associated with anti-Hu antibodies) and include peripheral neuropathy, limbic encephalitis, ataxia, as well as paraneoplastic chorea or optic neuritis [33]. Because anti-CRMP5 AAbs were found in almost 80% seropositive patients with lung cancer, CRMP5 has become an established biomarker for lung cancer-related paraneoplastic syndromes [33–35]. However, our CRMP5-seropositive patients did not have cancer at the time of testing.

In summary, our research identified novel AAbs specific against the optic nerve antigens unknown before in autoimmune neuro-retinopathy, suggesting the autoimmune nature of the syndrome. Indirect evidence, coming from clinical, functional, and imaging observations and immunology strongly suggest that these AAbs may play a direct role in the disease process. However, direct proof of the pathogenic potential of these anti-optic nerve antibodies is needed. If pathogenic, AAbs binding to proteins in optic nerve neurons may lead to disruption of protein function apoptosis of cells, as well as inhibiting enzymes that preserve neuronal glycolytic activity and neuronal integrity. Some of the neuronal antigens present in these patients coincide with molecular targets in other entities, such as NMO and other recognized paraneoplastic syndromes. These AAbs likely cause additional visual loss via an optic nerve-related component in autoimmune retinopathies that had been thus far underappreciated and underestimated in its frequency, and may offer additional treatment targets for patients with autoimmune neuro-retinopathies.

## References

[1] Adamus G, Brown L, Weleber RG (2009). Molecular biomarkers for autoimmune retinopathies: significance of anti-transducin-alpha autoantibodies. Exp Mol Pathol.

[2] Adamus G, Ren G, Weleber RG (2004). Autoantibodies against retinal proteins in paraneoplastic and autoimmune retinopathy. BMC Ophthalmol.

[3] Chan JW (2003). Paraneoplastic retinopathies and optic neuropathies. Sur Ophthalmol.

[4] Frohman L, Dellatorre K, Turbin R, Bielory L (2009). Clinical characteristics, diagnostic criteria and therapeutic outcomes in autoimmune optic neuropathy. Br J Ophthalmol.

[5] Damek DM (2005). Paraneoplastic retinopathy/optic neuropathy. Curr Treat Options Neurol.

[6] Goodwin J (2006). Autoimmune optic neuropathy. Curr Neurol Neurosci Rep.

[7] Radhakrishnan SS, Forma G, Carboni G, Mutolo MG, Adamus G, Iannaccone A (2010). Patterns of visual function loss in autoimmune neuro-retinopathy (AINR): psychophysical and electrophysiological findings. Invest Ophthalmol Vis Sci.

[8] Radhakrishnan SS, Laing AE, Adamus G, Iannaccone A (2009). Clinical characteristics of patients with auto-immune retinopathies (AIR) and neuropathies (AIN): differential diagnosis with retinitis pigmentosa (RP). Invest Ophthalmol Vis Sci.

[9] Wax MB, Yang J, Tezel G (2002). Autoantibodies in glaucoma. Cur Eye Res.

[10] Joachim SC, Pfeiffer N, Grus FH (2005). Autoantibodies in patients with glaucoma: a comparison of IgG serum antibodies against retinal, optic nerve, and optic nerve head antigens. Graefe's Arch Clin Exp Ophthalmol.

[11] Wax MB (2010) The case for autoimmunity in glaucoma. Exp Eye Res. doi:10.1016/j.exer.2010.08.016, Corrected Proof:1–4 10.1016/j.exer.2010.08.016 20801114

[12] Cross SA, Salomao D, Parisi J, Kryzer T, Bradley E, Mines J, Lam B (2003). VA L: paraneoplastic autoimmune optic neuritis with retinitis defined by CRMP-5-IgG. Ann Neurol.

[13] Lennon VA, Kryzer TJ, Pittock SJ, Verkman AS, Hinson SR (2005). IgG marker of optic-spinal multiple sclerosis binds to the aquaporin-4 water channel. J Exp Med.

[14] Adamus G (2009). Autoantibody targets and their cancer relationship in the pathogenicity of paraneoplastic retinopathy. Autoimmun Rev.

[15] Dutton JJ, Burde RM, Klingele TG (1982). Autoimmune retrobulbar optic neuritis. Am J Ophthalmol.

[16] Oyama Y, Burt RK, Thirkill C, Hanna E, Merrill K, Keltner J (2009). A case of autoimmune-related retinopathy and optic neuropathy syndrome treated by autologous nonmyeloablative hematopoietic stem cell transplantation. J Neuroophthalmol.

[17] Magrys A, Anekonda T, Ren G, Adamus G (2007). The role of anti-alpha-enolase autoantibodies in pathogenicity of autoimmune-mediated retinopathy. J Clin Immunol.

[18] Sirover MA (1999). New insights into an old protein: the functional diversity of mammalian glyceraldehyde-3-phosphate dehydrogenase. Biochim Biophys Acta.

[19] Gitlits VM, Sentry JW, Matthew ML, Smith AI, Toh BH (1997). Autoantibodies to evolutionary conserved epitopes of enolase in a patient with discoid lupus erythematosus. Immunol.

[20] Gitlits VM, Toh BH, Sentry JW (2001). Disease association, origin, and clinical relevance of autoantibodies to the glycolytic enzyme enolase. J Investig Med.

[21] Hsu SC, Molday RS (1990). Glyceraldehyde-3-phosphate dehydrogenase is a major protein associated with the plasma membrane of retinal photoreceptor outer segments. J Biol Chem.

[22] Dale RC, Candler PM, Church AJ, Wait R, Pocock JM, Giovannoni G (2006). Neuronal surface glycolytic enzymes are autoantigen targets in post-streptococcal autoimmune CNS disease. J Neuroimmunol.

[23] Adamus G, Aptsiauri N, Guy J, Heckenlively J, Flannery J, Hargrave PA (1996). The occurrence of serum autoantibodies against enolase in cancer-associated retinopathy. Cli Immunol Immunopath.

[24] Hernandez-Fonseca K, Massieu L (2005). Disruption of endoplasmic reticulum calcium stores is involved in neuronal death induced by glycolysis inhibition in cultured hippocampal neurons. J Neurosci Res.

[25] Butterfield DA, Hardas SS, Lange ML (2010). Oxidatively modified glyceraldehyde-3-phosphate dehydrogenase (GAPDH) and Alzheimer's disease: many pathways to neurodegeneration. J Alzheimers Dis.

[26] Kolln J, Ren HM, Da RR, Zhang Y, Spillner E, Olek M, Hermanowicz N, Hilgenberg LG, Smith MA, van den Noort S (2006). Triosephosphate isomerase- and glyceraldehyde-3-phosphate dehydrogenase-reactive autoantibodies in the cerebrospinal fluid of patients with multiple sclerosis. J Immunol.

[27] Oldstone MB (2005). Molecular mimicry, microbial infection, and autoimmune disease: evolution of the concept. Curr Top Microbiol Immunol.

[28] Pancholi V (2001). Multifunctional alpha-enolase: its role in diseases. Cell Mol Life Sci.

[29] Marnetto F, Hellias B, Granieri L, Frau J, Patanella AK, Nytrova P, Sala A, Capobianco M, Gilli F, Bertolotto A (2009). Western blot analysis for the detection of serum antibodies recognizing linear Aquaporin-4 epitopes in patients with Neuromyelitis Optica. J Neuroimmunol.

[30] Haase CG, Schmidt S (2001). Detection of brain-specific autoantibodies to myelin oligodendrocyte glycoprotein, S100[beta] and myelin basic protein in patients with Devic's neuromyelitis optica. Neurosci Lett.

[31] Marignier R, Nicolle A, Watrin C, Touret M, Cavagna S, Varrin-Doyer M, Cavillon G, Rogemond V, Confavreux C, Honnorat J (2010). Oligodendrocytes are damaged by neuromyelitis optica immunoglobulin G via astrocyte injury. Brain.

[32] Waters P, Vincent A (2008). Detection of anti-aquaporin-4 antibodies in neuromyelitis optica: current status of the assays. Int MS J.

[33] Yu Z, Kryzer TJ, Griesmann GE, Kim K, Benarroch EE, Lennon VA (2001). CRMP-5 neuronal autoantibody: marker of lung cancer and thymoma-related autoimmunity. Ann Neurol.

[34] Pittock SJ, Kryzer TJ, Lennon VA (2004). Paraneoplastic antibodies coexist and predict cancer, not neurological syndrome. Ann Neurol.

[35] Margolin E, Flint A, Trobe JD (2008). High-titer collapsin response-mediating protein-associated (CRMP-5) paraneoplastic optic neuropathy and vitritis as the only clinical manifestations in a patient with small cell lung carcinoma. J Neuroophthalmol.

